# Cardiopulmonary-related patient-reported outcomes in a randomized clinical trial of radiation therapy for breast cancer

**DOI:** 10.1186/s12885-021-08916-z

**Published:** 2021-11-04

**Authors:** Hilde Van Parijs, Vincent Vinh-Hung, Christel Fontaine, Guy Storme, Claire Verschraegen, Dung M. Nguyen, Nele Adriaenssens, Nam P. Nguyen, Olena Gorobets, Mark De Ridder

**Affiliations:** 1 Universitair Ziekenhuis Brussel, Vrije Universiteit Brussel, 101 Laarbeeklaan, 1090 Brussels, Belgium; 2 Centre Hospitalier Universitaire (CHU) de Martinique, Fort-de-France, France; 3 Ohio State University, Columbus, OH USA; 4 School of Medicine, Vietnam National University, Ho Chi Minh City, Vietnam; 5 Howard University, Washington, DC USA; 6 Ukrainian Military Medical Academy, Kiev, Ukraine

**Keywords:** Quality of life, Patient reported outcome measures, Dyspnea, Fatigue, Pain

## Abstract

**Background:**

Long-term prospective patient-reported outcomes (PRO) after breast cancer adjuvant radiotherapy is scarce. TomoBreast compared conventional radiotherapy (CR) with tomotherapy (TT), on the hypothesis that TT might reduce lung-heart toxicity.

**Methods:**

Among 123 women consenting to participate, 64 were randomized to CR, 59 to TT. CR delivered 50 Gy in 25 fractions/5 weeks to breast/chest wall and regional nodes if node-positive, with a sequential boost (16 Gy/8 fractions/1.6 weeks) after lumpectomy. TT delivered 42 Gy/15 fractions/3 weeks to breast/chest wall and regional nodes if node-positive, 51 Gy simultaneous-integrated-boost in patients with lumpectomy. PRO were assessed using the European Organization for Research and Treatment of Cancer questionnaire QLQ-C30. PRO scores were converted into a symptom-free scale, 100 indicating a fully symptom-free score, 0 indicating total loss of freedom from symptom. Changes of PRO over time were analyzed using the linear mixed-effect model. Survival analysis computed time to > 10% PRO-deterioration. A post-hoc cardiorespiratory outcome was defined as deterioration in any of dyspnea, fatigue, physical functioning, or pain.

**Results:**

At 10.4 years median follow-up, patients returned on average 9 questionnaires/patient, providing a total of 1139 PRO records. Item completeness was 96.6%. Missingness did not differ between the randomization arms. The PRO at baseline were below the nominal 100% symptom-free score, notably the mean fatigue-free score was 64.8% vs. 69.6%, pain-free was 75.4% vs. 75.3%, and dyspnea-free was 84.8% vs. 88.5%, in the TT vs. CR arm, respectively, although the differences were not significant. By mixed-effect modeling on early ≤2 years assessment, all three scores deteriorated, significantly for fatigue, *P* ≤ 0.01, without effect of randomization arm. By modeling on late assessment beyond 2 years, TT versus CR was not significantly associated with changes of fatigue-free or pain-free scores but was associated with a significant 8.9% improvement of freedom from dyspnea, *P* = 0.035. By survival analysis of the time to PRO deterioration, TT improved 10-year survival free of cardiorespiratory deterioration from 66.9% with CR to 84.5% with TT, *P* = 0.029.

**Conclusion:**

Modern radiation therapy can significantly improve long-term PRO.

**Trial registration:**

Trial registration number ClinicalTrials.govNCT00459628, April 12, 2007 prospectively.

**Supplementary Information:**

The online version contains supplementary material available at 10.1186/s12885-021-08916-z.

## Background

Breast cancer is the most common cancer among women worldwide, and the second most frequent cause of cancer death in more developed regions [1]. It is a major contributor to the high overall cancer disability-adjusted life-years in very high Human Development Index countries, with quite a large contribution of years spent with a disability [2]. Radiotherapy improves tumor control and survival in breast cancer [3, 4]. With improved survival, quality of life (QOL) is becoming increasingly important. Treatment toxicities adversely affect QOL, and radiation therapy has been specifically associated with increased risks of heart disease and radiation pneumonitis [5]. As radiation techniques evolve continuously [6], there is need to evaluate whether breast cancer patients can benefit from new technologies.

Tomotherapy is a treatment system which provides intensity modulated and volumetric image guidance radiation therapy (IMRT-IGRT) [7, 8]. The irradiation is delivered helicoidally providing highly conformal shaping of dose distribution. Integrated imaging improves the accuracy of the treatment, allowing to treat tumors yet sparing critical structures. TomoBreast is a randomized clinical trial that investigates whether the technical advantage of tomotherapy translates into a substantial reduction of pulmonary and cardiac toxicities, as compared with conventional radiotherapy [9]. Previous reports of the trial have shown that tomotherapy improved the homogeneity of the dose to targets, decreased the dose to the heart and ipsilateral lung, and reduced the pooled all-grades lung-heart toxicity [10]. Subsequent analyses of the trial’s data established that lung function declined during the initial 3 months more markedly in the conventional radiotherapy arm and continued to decline thereafter [11]. Thus, at the very least, the trial already showed that lung toxicity is detectable early on and is affected by the choice of radiation technique. Previous preliminary analysis of the trial’s QOL data at 2 years further suggested an improvement of global health status and faster recovery from fatigue with tomotherapy [12]. With continued follow-up that reached 10 years, the present study seeks to assess the long-term impact of the trial on respiratory-related patient-reported outcomes (PRO).

## Methods

### Study design and patients

TomoBreast is a single center phase III randomized controlled trial comparing accelerated adjuvant radiotherapy with the tomotherapy system (TT), versus conventional post-surgery radiotherapy (CR) for breast cancer. The trial was conducted in 2007–2011 at the Universitair Ziekenhuis Brussel (UZ Brussel), Belgium. The trial tested the hypothesis that TT treatment, as compared with CR, could substantially reduce the incidence of pulmonary and cardiac toxicities (primary outcome), without increase of recurrences (secondary outcome). Pulmonary and cardiac toxicities were to be assessed by medical imaging and functional tests. Medical imaging was not implemented for lack of funding. Functional assessment was implemented under the form of five parallel modules: 1) echocardiographic evaluation under cardiologist guidance [13]; 2) pulmonary function test managed by the pneumology department [11]; 3) shoulder-arm physical evaluation managed by the physiotherapy unit [14]; 4) oncologist’s recording of clinical toxicities using the Late Effects Normal Tissues - Subjective, Objective, Management, Analytic (LENT-SOMA), and the Radiation Therapy Oncology Group (RTOG) scores [15]; and 5) patient-reported outcomes (PRO) as will be detailed in the next section. Analyses of the echocardiography, pulmonary function, physical evaluation, and clinician’s assessed toxicities limited to five-years curated data are on-going. The present study assesses exclusively the cardiopulmonary-related PRO extending over 10 years.

Eligible patients were women ≥18 years old with histologically proven stage I or II (T1-3N0 or T1-2N1 M0) invasive breast carcinoma [16], who had surgery (lumpectomy or mastectomy) with clear resection margins. Exclusion criteria were prior breast or thoracic radiotherapy, pregnancy, lactation, psychiatric or addictive disorders, and fertile patients without effective contraception. Patients who gave written informed consent were randomized to either CR (control arm), or TT (experimental arm). CR used the UZ-Brussel standard procedure of tangential chest fields, with an additional supraclavicular field in the case of nodal involvement, with a dose-fractionation of 50 Gy in 25 fractions/5 weeks, and a sequential electron boost of 16 Gy in 8 fractions/2 weeks in the case of breast-conserving surgery. CR planning used forward field-in-field intensity-modulated radiation treatment. TT used the Tomotherapy system. Target areas (breast for conservative surgery, thorax wall for mastectomy, plus nodal areas in node-positive patients) were treated with a dose-fractionation of 42 Gy in 15 fractions/3 weeks, and with a simultaneous integrated boost of 0.6 Gy/fraction in the case of breast-conserving surgery. TT-planning used the procedure “Tomo supine” for helical tomotherapy [17].

The study size required a minimum of 118 patients, computed on the hypothesis that TT would reduce the incidence of lung-heart any-grade toxicity from 25% with CR to 5% with TT, by two-sided testing with a power of 0.80 at a significance level of 0.05. Randomization was balanced by nodal status, type of surgery, and chemotherapy sequence using Efron’s biased coin method [18]. The randomization was conducted by a data manager independently of the clinicians. The patients and the clinical staff were not blinded to the allocation but had no influence on the random drawing process.

### QOL assessments

PRO measures were assessed using the European Organization for Research and Treatment of Cancer (EORTC) core questionnaire (QLQ-C30). The breast module QLQ-BR23 was collected but not used in the present study. The present study retained the QLQ-C30’s five multi-item functional scales (physical, role, cognitive, emotional, and social), and three symptom scales (fatigue, pain, and dyspnea), as well as a global health scale [19]. The items were rated by patients using a seven-point response from 1 (“very poor”) to 7 (“excellent”) for global health status items #29 and #30, and a four-point response from 1 (“not at all”) to 4 (“very much”) for other items. The scale and item scores were linearly transformed to a 0–100 range. Functional and symptom scales were recoded such that a higher score represented a better level of functioning and symptom-free state. An overall summary measure labelled “C30 summary”, was also computed [20].

The Dutch or the French version of the QLQ-C30 printed questionnaire was used, in accordance with each patient’s preference. The questionnaires were collected before radiotherapy (baseline), at the last session of radiotherapy, at 1–3 months after completion of radiotherapy, and thereafter once yearly until February 2019.

### Statistical analyses

The linear mixed effects model and specific PRO deterioration free survival were used for the PRO data analyses, as detailed below.

The linear mixed effects model fitted the PRO measures expressed as percent change from each patient’s baseline PRO. Time was modeled as a random effect and therapy as a fixed group effect. Coefficients were estimated through maximum likelihood [21]. The linear mixed effects were modeled on the full follow-up, then further modeled by period, early (assessments ≤2 years from randomization), and late (> 2 years from randomization).

Specific PRO survival estimates considered the time to event, in which event was defined as the degradation of a QOL scale to below 10% from baseline [22]. The 10% cutoff is analogous to the minimally important difference (MID) [23–27]. However, the data at hand was not used to determine a MID. The rationale is to apply a common cutoff applicable to future studies comparing the PRO to echocardiography and pulmonary function tests, taking into consideration that 10% change is concordant with the variable precision of these exams [13, 28]. The time-to-deterioration values for each individual patient were computed with the constraint that the linear regression of the patient’s QOL degradation over time should be significant at a 0.05 level. The patients were censored at the last follow-up time or at time of death. The Kaplan-Meier method and log-rank tests were applied [22]. In addition to the pre-defined QOL scales, a post-hoc “cardiorespiratory-related” composite event was defined as deterioration in any of the dyspnea, physical functioning, fatigue, and/or pain measures.

All analyses were done by intent-to-treat. No patient was excluded. Computations used R version 3.5.2 [29]. The specific R packages and functions used were: “tableone” for tables’ layout, using the Student’s t-test for the comparison of means and the chi-square test for the comparison of proportions; “PROscorerTools” for computation of the QOL scales; “survival” for the implementation of the Kaplan-Meier time-to-event analysis and the log-rank test [30]; the function “kmplot” for survival plot layout; and “lme4” for the linear mixed effects model [21]. Implementation of time-to-deterioration used an in-house script, available on request. Missing data were handled by listwise deletion.

## Ethical statement

This trial complied fully with guidelines for Good Clinical Practice and the Declaration of Helsinki. Written informed consent was obtained from each patient. The trial was approved by the ethics committee of the Universitair Ziekenhuis Brussel (UZ-Brussel), and was registered on ClinicalTrials.gov, number NCT00459628.

## Results

### Baseline characteristics

**Fig. 1:**
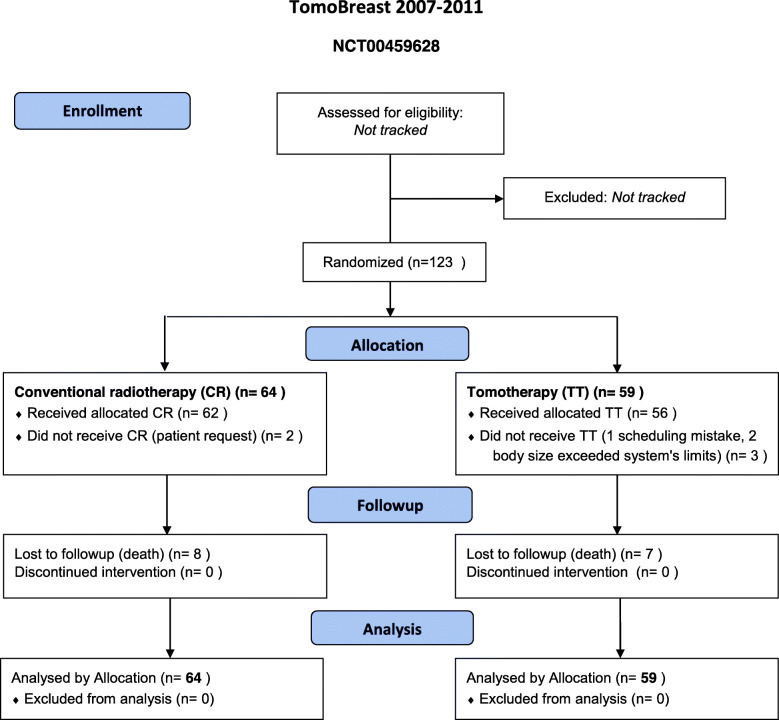
Consort 2010 flow diagram of the TomoBreast trial

The trial, conducted at the UZ-Brussel, started in May 2007 and closed in July 2011 when the accrual was reached. A total of 123 women consented to participate (Fig. 1). Of these, 64 (52%) were randomized to CR and 59 (48%) to TT. Of the 64 patients allocated to CR, 2 received TT by request. Of the 59 patients allocated to TT, 3 received CR, 1 because of an appointment scheduling error, 2 because tomotherapy was unsuitable due to the patient’s body size exceeding the system’s limits.

The patients’ characteristics showed some imbalances between the two arms. There were non-significantly more smokers, more nodal disease, more concurrent chemo-radiation, and significantly more axillary lymph node dissection (*P* = 0.043), and HER2 overexpression and trastuzumab therapy (*P* = 0.055) in the TT group (Table 1).

**Table 1:** Patient characteristics

	Level	Conventional Radiotherapy	Hypofractionated Tomotherapy	p
		***N*** = 64	***N*** = 59	
Age, years: mean (sd)		57.8 (11.6)	55.1 (11.5)	0.198
Karnofsky Performance Status: mean (sd)		94.1 (8.4)	94.7 (7.2)	0.678
Body Mass Index kg/m^2^: mean (sd)		25.7 (4.2)	26.0 (5.4)	0.720
Smoker: N (%)	No	46 (72)	38 (64)	0.493
	Yes	8 (12)	12 (20)	
	Ex-smoker	10 (16)	9 (15)	
Laterality: N (%)	Right	31 (48)	24 (41)	0.402
	Left	32 (50)	35 (59)	
	Bilateral	1 (2)	0 (0)	
Grade: N (%)	1	18 (30)	16 (28)	0.549
	2	25 (42)	29 (51)	
	3	17 (28)	12 (21)	
Stage: N (%)	I	28 (44)	25 (42)	0.577
	IIA	31 (48)	26 (44)	
	IIB	5 (8)	8 (14)	
Tumor Size mm: mean (sd)		19.8 (11.0)	20.3 (11.6)	0.820
Nodal Status: N (%)	Negative	48 (75)	38 (64)	0.279
	Positive	16 (25)	21 (36)	
ER: N (%)	Negative	8 (12)	11 (19)	0.489
	Positive	56 (88)	48 (81)	
PR: N (%)	Negative	18 (28)	13 (22)	0.569
	Positive	46 (72)	46 (78)	
HER2 FISH-amplified: N (%)	No	61 (95)	47 (82)	0.047
	Yes	3 (5)	10 (18)	
Mastectomy: N (%)	No	45 (70)	33 (56)	0.142
	Yes	19 (30)	26 (44)	
Axillary Lymph Node Dissection: N (%)	No [=SN only]	45 (70)	30 (51)	0.043
	Yes	19 (30) [9 after SN]	29 (49) [13 after SN]	
Chemotherapy Schedule: N (%)	None planned	38 (59)	29 (49)	0.499
	Prior to RT	7 (11)	7 (12)	
	Concomitant	19 (30)	23 (39)	
Hormone therapy: N (%)	No	9 (14)	8 (14)	0.155
	Tamoxifen	26 (41)	16 (27)	
	Letrozole	26 (41)	26 (44)	
	Goserelin(*)	3 (5)	9 (15)	
Trastuzumab: N (%)	No	61 (95)	49 (83)	0.055
	Yes	3 (5)	10 (17)	
Nodal Radiotherapy: N (%)	No	48 (75)	39 (66)	0.376
	Yes	16 (25)	20 (34)	

SN: sentinel lymph nodes biopsy. (*) Goserelin with or without tamoxifen or letrozole

As of February 4, 2020, the median follow-up of patients alive was 10.4 years. A total of 95 patients had no disease-related events and 28 had one or more events: 15 deaths, 1 local recurrence, 0 nodal recurrence, 14 metastases (either from a primary breast tumor or from a new primary tumor), and 13 new primary tumors. The locations of the new primary tumors were: 4 contralateral breast, of which 3 were invasive and 1 was non-invasive; 3 colorectal; 1 bladder; 1 kidney; 1 ovary; 1 lung; 1 skin basal cell carcinoma; and 1 skin basal cell and squamous cell carcinoma. The overall survival and the disease-free survival did not differ between the two groups, *P* = 0.971 and *P* = 0.569, respectively (Supplementary eFigure F1).

### Completeness of assessments

The QOL questionnaires were continuously collected yearly. The patients returned on average 9 questionnaires (median = 10, inter-quartile range = 8–11). The time span covered by the collected questionnaires ranged from 0.5 to 11.3 years from randomization, averaging 8.1 years (median 8.5, inter-quartile range 7.2–9.9 years) (Supplementary eFigure F2). The overall percentage of missing items within the collected questionnaires was 3.4% and the rate of completed items was 96.6%. Number of questionnaires, follow-up duration, and pattern of missing data did not differ by randomization arm (Supplementary eFigure F3).

### QOL outcomes

The patients presented with a deteriorated baseline QOL. The average score was below 100 by more than 10 points in all measures. The low baseline values –attributable to the post-surgery status– were comparable between the two randomization arms (Table 2).

**Table 2:** Baseline patient-reported outcome measures

	Conventional Radiotherapy		Hypofractionated Tomotherapy		P
Measure	N = 64		N = 59		
	Mean	SD	Mean	SD	
Global health status	68.6	(21.5)	67.2	(17.5)	0.697
C30 summary	82.1	(14.0)	80.6	(13.3)	0.537
Physical functioning	84.4	(18.5)	83.2	(16.0)	0.700
Role functioning	69.5	(27.3)	66.4	(29.3)	0.539
Emotional functioning	78.3	(18.3)	74.4	(20.0)	0.265
Cognitive functioning	86.5	(20.3)	82.8	(22.3)	0.339
Social functioning	80.5	(22.3)	81.6	(20.7)	0.764
Fatigue free	69.6	(20.7)	64.8	(24.9)	0.242
Pain free	75.3	(24.3)	75.4	(24.2)	0.970
Dyspnea free	88.5	(22.4)	84.8	(26.5)	0.391

Rapid improvement over the first 1–3 years was observed in almost all QOL measures, most notably in global health status, role and social functioning, fatigue, and pain (figure not shown). Most measures appeared to plateau thereafter.

Fitting the PRO with the linear mixed effects models showed an improvement with time in all measures (Supplementary eTable T2). In addition, tomotherapy as compared with conventional radiotherapy was associated with a trend towards lower rates of dyspnea (4.1%), *P* = 0.090. Modeling the mixed effects according to the early (≤2 years from randomization) and late (> 2 years from randomization) period of assessment showed that the largest improvements in PROs occurred early. Regarding the time effect, significant or nearly significant improvements were observed in the early period in global health status (*P* = 0.018), physical functioning (*P* = 0.091), role functioning (*P* = 0.001), social functioning (*P* = 0.004), and fatigue (*P* = 0.006) (Table 3, column Time Early effect). There were no significant time effects in the late period. Regarding the randomization group effect, tomotherapy was associated with a significantly poorer global health status in the early period (*P* = 0.032), but not in the late period. Better freedom from dyspnea was significantly associated with tomotherapy in the late period, *P* = 0.035 (Table 3, column TT Late effect). Tomotherapy was also associated with better cognitive functioning, *P* < 0.001 (Table 3, column TT Late effect).

**Table 3:** Linear mixed model by early and by late assessment period

	Early (≤2 years) assessment		Late (> 2 years) assessment	
	Time Early effect coef/year	TT Early effect coef TT	Time Late effect coef/year	TT Late effect coef TT
Global health status	11.3 *	−7.4 *	−0.9	−5.7
C30 summary	5.8 **	−1.2	−0.6	1.8
Physical functioning	5.3 °	0.3	−0.7	−2.4
Role functioning	25.0 ***	−4.5	−1.3	5.0
Emotional functioning	2.3	0.7	−0.7	5.2
Cognitive functioning	3.6	3.1	−1.5 *	15.9 ***
Social functioning	13.4 **	−2.1	−1.1	−8.1
Fatigue free	13.6 **	−1.6	−0.5	2.2
Pain free	10.8	−4.6	−0.3	−1.8
Dyspnea free	4.8	0.8	−1.1	8.9 *

Linear mixed model, effect of time and tomotherapy (TT, versus conventional radiotherapy) on patient reported outcome measure by early and by late assessment period. *P*-values: ° ≤ 0.10; * ≤ 0.05; ** ≤ 0.01; *** ≤ 0.001. Coef: model’s coefficient of percent change relative to baseline

**Fig. 2:**
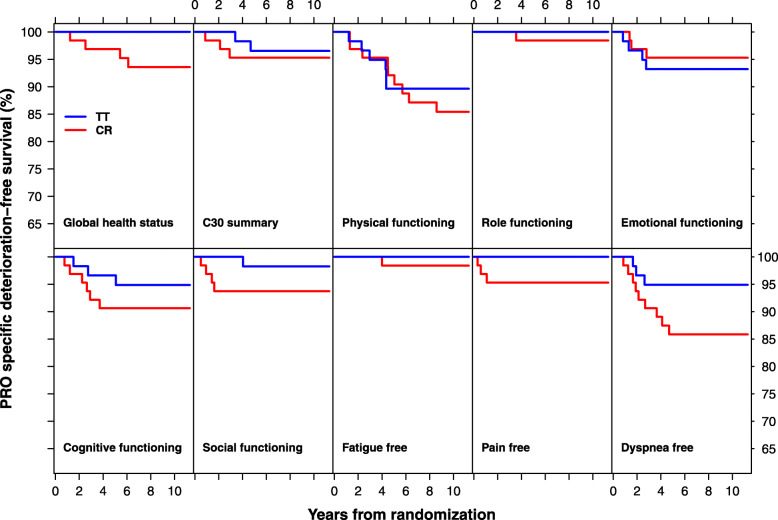
Patient reported outcome (PRO) specific deterioration free survival

Specific QOL deterioration free survival found a poorer survival free from dyspnea in the CR group as compared with TT, log-rank test *P* = 0.098 (Table 4). The 10–year dyspnea free survival estimate was 85.9% (95%CI: 77.7–94.9%) in the CR arm, as compared with 94.9% (89.5–100.0%) in the TT arm. Specific survival free from deterioration of the Global health status was significantly poorer in the CR group, 93.6% at 10 years, as compared with 100% in the TT arm, *P* = 0.052. Despite lack of significance in the other QOL scales, the survival plots showed moreover a trend of deterioration free survival in favor of TT, notably regarding Cognitive functioning, Social functioning, and Pain free (Fig. 2).

**Table 4:** Patient reported outcome (PRO) specific deterioration free survival (SDFS) estimated at 10 years

	Conventional Radiotherapy		Hypofractionated Tomotherapy		Log-rank P
PRO scale	10-year SDFS	(95% CI)	10-year SDFS	(95% CI)	
Global health status	93.6	(87.7–99.9)	100	(100–100)	0.052
C30 summary	95.3	(90.3–100)	96.5	(92.0–100)	0.701
Physical functioning	85.4	(77.1–94.7)	89.6	(82.1–97.8)	0.558
Role functioning	98.4	(95.4–100)	100	(100–100)	0.337
Emotional functioning	95.3	(90.3–100)	93.2	(87.0–99.9)	0.609
Cognitive functioning	90.6	(83.8–98.1)	94.9	(89.3–100)	0.355
Social functioning	93.8	(88.0–99.9)	98.2	(94.9–100)	0.201
Fatigue free	98.4	(95.4–100)	100	(100–100)	0.342
Pain free	95.3	(90.3–100)	100	(100–100)	0.094
Dyspnea free	85.9	(77.7–94.9)	94.9	(89.5–100)	0.098

**Fig. 3:**
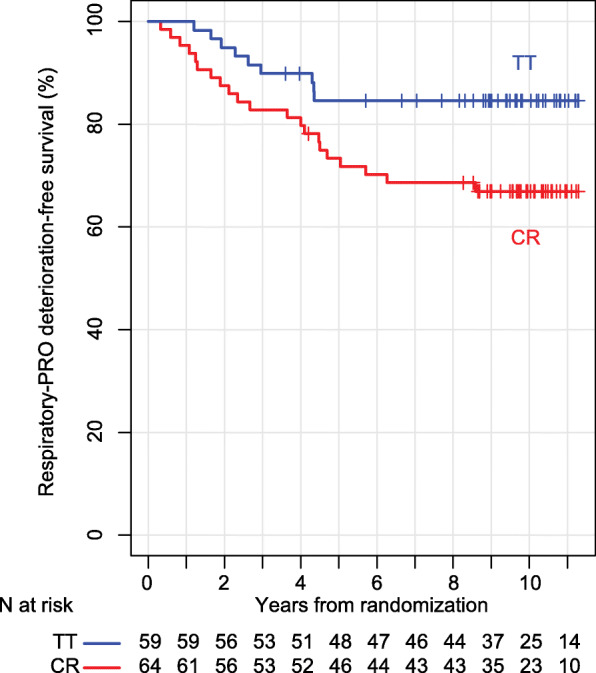
Patient reported outcome (PRO) specific survival free from deterioration in any of dyspnea, fatigue, pain, or physical functioning scales, by randomization arm. TT: tomotherapy. CR: conventional radiotherapy

In the post-hoc analysis using the composite QOL outcome combining the pain measure with any of dyspnea, physical functioning, or fatigue scales, the specific survival free from deterioration in the composite outcome was significantly improved with TT arm, log-rank *P* = 0.029. Survival plot showed a clear separation in favor of TT (Fig. 3). The estimated 10-year survival free of deterioration was 84.5% (95%CI: 75.7–94.4%) in the TT arm, as compared with 66.9% (95%CI: 56.2–79.6%) in the CR arm.

## Discussion

The improved long-term cardiorespiratory-related outcome in the tomotherapy arm is a key finding of the study, remarkably considering the high proportion of patients receiving concurrent chemotherapy and trastuzumab, and the high number of patients who were current or ex-smokers receiving lymph node irradiation. Previously, the diffusing capacity of the lung for carbon monoxide (DLCO) at 3 years was shown to be significantly better with tomotherapy [10, 31]. At 12 years follow-up, the respiratory-sparing effect of tomotherapy has been borne out through patient-reported outcome measures. These observations have an important implication: toxicity is detectable early, the impact can be long lasting, applying advanced radiotherapy techniques to spare the lungs and heart is of foremost concern.

Why should lung and heart toxicities be considered together, and why should fatigue and pain be included with dyspnea as cardiopulmonary outcomes? Both the lungs and heart are central to oxygen uptake and transport. Physical fitness requires an adequate supply of oxygen, which is dependent on a coordinated chain of processes that include ventilation, pulmonary blood flow, gas exchange, and cardiac output [32]. Disturbances in these processes due to cardiac or pulmonary impairment, aging, or disease, iatrogenic or not, could manifest as symptoms of decreased exercise tolerance, or increased fatigue and breathlessness [33]. Dyspnea is the most prevalent symptom among patients with cardiac and respiratory diseases [34]. However, dyspnea can be masked. Self-reported breathlessness can decrease with age, sensitivity to alteration of lung function can differ among patients, and the perception of dyspnea can be blunted in the course of respiratory and heart diseases [35].

Next to dyspnea, chest pain is a chief complaint in acute and long-term heart disease and is also common in patients with lung disease [36]. However, the chest is not the sole pain location. Non-chest pain is prevalent in patients with myocardial infarction and in heart failure [37] . Likewise, a high prevalence of bodily pain has been reported in chronic obstructive pulmonary disease [38].

TomoBreast used a composite lung and heart outcome in light of the trial size and the pragmatic consideration that cardiac events in modern radiotherapy are rare. The choice is validated by physiology, clinical pathology, and the well-documented overlap of cardiopulmonary symptoms. Furthermore, the combination of pain, fatigue, physical functioning, and dyspnea measures derived from the QLQ-C30 mirrors specific instruments for the measurement of lung and heart outcomes [39]. None of the symptoms is specific. Pain arising in a breast cancer can have many causes, not only from the breast, muscles, nerves, bone, but also from heart or lung. There is no specificity. Nevertheless, combined with other symptoms, the constellation improves the sensitivity to detect a substantial impact on patient quality of life. To our knowledge, this study is the first that explores the QLQ-C30 items in a cardiopulmonary perspective.

The study has limitations. The small number of patients allowed no subgroup analysis; neither by chemotherapy nor by regional nodal irradiation. Stratification did not consider trastuzumab treatment. The linear mixed models were not established in advance. The criteria of QOL deterioration were not prespecified. The study did not consider the precision of the QOL measurements. Single-item symptom scales were limited to a range of 1 to 4 possible responses. Conversion to a 0–100 range translates to only four possible values, 0, 33.3, 66.7, and 100, far from the precision implied by the need to detect 10% changes. Cardiopulmonary-related symptoms were not complemented with additional specific patient-reported lung or heart outcome measures such as cough and sputum, edema, palpitations, dizziness, or syncope. Tomotherapy patients reported less deterioration in measures of fatigue, pain, and dyspnea. We ascribe this to better lung-heart sparing. However, we cannot exclude that the favorable tomotherapy outcome could result from non-cardiopulmonary mechanisms.

Counterbalancing the limitations, the study argues against practice bounds to the development of breast radiotherapy. Conventional radiotherapy of the breast is still the preferred technique, advanced radiation is discouraged from reimbursement [9]. The present study is the counterpoint. It shows that an advanced technique can provide a meaningful long-term improvement in patient-reported outcomes.

The control arm and the experimental arm fractionation schedules differed, which might be perceived as a confounding weakness – if one discards all current evidence of large prospective randomized trials showing that moderate hypofractionation does not affect the outcome of breast cancer [40, 41]. Using normofractionation in the control arm maintained continuity with the majority of historic trials that demonstrated a survival advantage with breast radiotherapy [42]. Using hypofractionation in the experimental arm bridges with today’s practice. With hindsight, TomoBreast was designed against obsolescence of fractionation.

In summary, improved cardiorespiratory-related outcome in tomotherapy patients is a proof of concept that advanced radiation techniques can have a substantial clinical impact. The reduction of lung and heart toxicities is detectable early [31]. In the long-term, this translates into a significant advantage in patient self-reported outcome. Investing in lung/heart-sparing techniques do yield a benefit. Currently many approaches are available [43]. The challenge will be to choose the most cost-effective technique applicable to the largest number of patients.

## Conclusions

Hypofractionated tomotherapy was associated with significantly better long-term survival-free from cardiorespiratory-related deterioration. The study is a proof of concept that patient-reported outcome might be improved using advanced radiation techniques.

## Supplementary Information

**Additional file 1.**

## Data Availability

Data are available on request to Hilde Van Parijs, email Hilde.VanParijs@uzbrussel.be, and Vincent Vinh-Hung, email vh@onco.be. Protocol uploaded with manuscript.

## Abbreviations

CR: control arm
DLCO: diffusing capacity of the lung for carbon monoxide
EORTC: European Organization for Research and Treatment of Cancer
IMRT-IGRT: intensity modulated and volumetric image guidance radiation therapy
PRO: patient-reported outcome
QLQ-BR23: breast module
QLQ-C30: core questionnaire
QOL: quality of life
TT: experimental arm
UZ-Brussel: Universitair Ziekenhuis Brussel
