# Supplementary material for: Cardiopulmonary-related patient-reported outcomes in a randomized clinical trial of radiation therapy for breast cancer
Source: BMC Cancer. 2021 Nov 4;21:1177. doi: 10.1186/s12885-021-08916-z (PMC8569957; doi:10.1186/s12885-021-08916-z)
Supplement: Supplementary file 1 — Additional file 1. [file 12885_2021_8916_MOESM1_ESM.docx]

# Supplementary Online Content

Cardiopulmonary-related patient-reported outcome in a randomized clinical trial of radiation therapy for breast cancer.

**eFigures**

- **eFigure F1.** Overall and disease-free survival by randomization arm.
- **eFigure F2.** Distribution of patients' quality of life (QOL) assessment timepoints.
- **eFigure F3.** Missing QOL item responses by randomization arm.

**eTables**

- **eTable T1.** Cumulative distribution of the number of assessments.
- **eTable T2.** Linear mixed model, all periods.
- **eTable T3.** Time to deterioration analysis: Risks of patient-reported outcome deterioration by 10% at 10 years.

# eFigures

## eFigure F1. Overall and disease-free survival by randomization arm. CR: conventional radiotherapy. TT: hypofractionated tomotherapy.

## eFigure F2. Distribution of patients' quality of life (QOL) assessment timepoints. CR: conventional radiotherapy. TT: tomotherapy.

## eFigure F3. Missing QOL item responses by randomization arm. CR: conventional radiotherapy. TT: tomotherapy.

# eTables.

## eTable T1. Cumulative distribution of the number of assessments.

CR: conventional radiotherapy. TT: tomotherapy.

| **Number of assessments** | **All**  **N (%)** | **CR**  **N (%)** | **TT**  **N (%)** |
| --- | --- | --- | --- |
| 5 | 119 (96.7) | 61 (95.3) | 58 (98.3) |
| 6 | 114 (92.7) | 59 (92.2) | 55 (93.2) |
| 7 | 108 (87.8) | 55 (85.9) | 53 (89.8) |
| 8 | 102 (82.9) | 53 (82.8) | 49 (83.1) |
| 9 | 88 (71.5) | 48 (75) | 40 (67.8) |
| 10 | 71 (57.7) | 36 (56.3) | 35 (59.3) |
| 11 | 43 (35) | 22 (34.4) | 21 (35.6) |
| 12 | 6 (4.9) | 3 (4.7) | 3 (5.1) |

## eTable T2. Linear mixed model, all periods.

Effect of Time: the coefficient indicates the percent change in QOL per year relative to baseline. Effect of Therapy: the coefficient indicates the change in QOL attributable to tomotherapy (TT) versus conventional radiotherapy. P-values: ° ≤ 0.10; * ≤ 0.05; ** ≤ 0.01; *** ≤ 0.001.

| QOL Measure | Time Effect coefficient/year |  | Therapy Effect  coefficient TT |  |
| --- | --- | --- | --- | --- |
| Global health status | 1.4 |  | −6.2 | * |
| C30 summary | 0.8 |  | −0.3 |  |
| Physical functioning | 0.8 |  | −0.6 |  |
| Role functioning | 2.9 | * | −2.5 |  |
| Emotional functioning | 0.5 |  | 2.0 |  |
| Cognitive functioning | −0.3 |  | 9.1 | *** |
| Social functioning | 2.1 | * | −3.7 |  |
| Fatigue free | 3.3 | ** | 0.2 |  |
| Pain free | 1.6 |  | −4.5 |  |
| Dyspnea free | 0.0 |  | 4.1 | ° |

## eTable T3. Patient reported outcome (PRO) specific deterioration free survival (SDFS) estimated at 10 years.

|  | **Conventional Radiotherapy** | | **Hypofractionated Tomotherapy** | | Log-rank P |
| --- | --- | --- | --- | --- | --- |
| PRO scale | 10-year SDFS | (95% CI) | 10-year SDFS | (95% CI) |  |
| Global health status | 93.6 | (87.7–99.9) | 100 | (100–100) | 0.052 |
| C30 summary | 95.3 | (90.3–100) | 96.5 | (92.0–100) | 0.701 |
| Physical functioning | 85.4 | (77.1–94.7) | 89.6 | (82.1–97.8) | 0.558 |
| Role functioning | 98.4 | (95.4–100) | 100 | (100–100) | 0.337 |
| Emotional functioning | 95.3 | (90.3–100) | 93.2 | (87.0–99.9) | 0.609 |
| Cognitive functioning | 90.6 | (83.8–98.1) | 94.9 | (89.3–100) | 0.355 |
| Social functioning | 93.8 | (88.0–99.9) | 98.2 | (94.9–100) | 0.201 |
| Fatigue free | 98.4 | (95.4–100) | 100 | (100–100) | 0.342 |
| Pain free | 95.3 | (90.3–100) | 100 | (100–100) | 0.094 |
| Dyspnea free | 85.9 | (77.7–94.9) | 94.9 | (89.5–100) | 0.098 |
